# Cerclage for singleton pregnancies with an extremely short cervix (≤10 mm) and no history of spontaneous preterm birth: A multisite observational study

**DOI:** 10.1002/pmf2.70262

**Published:** 2026-03-11

**Authors:** Namita Kansal, Meralis Lantigua‐Martinez, Steven Friedman, Sonia Khurana, Cody Goldberger, Erinn M. Hade, Jenna Silverstein, Dana Berger, Ashley S. Roman, Justin S. Brandt, Christina A. Penfield

**Affiliations:** ^1^ Division of Maternal‐Fetal Medicine Department of Obstetrics and Gynecology NYU Grossman School of Medicine New York New York USA; ^2^ Division of Biostatistics Department of Population Health NYU Grossman School of Medicine New York New York USA; ^3^ Division of Maternal‐Fetal Medicine Department of Obstetrics and Gynecology Sisters of Charity Hospital Buffalo New York USA; ^4^ Department of Obstetrics and Gynecology NYU Langone Health New York New York USA

**Keywords:** cerclage, pregnancy latency term delivery tended to occur more frequently in patients who received a cerclage compared to those who did not, preterm birth, short cervix

## Abstract

**Introduction:**

There is uncertainty about the benefits of cerclage in patients with transvaginal cervical length (TVCL) ≤10 mm and no prior spontaneous preterm birth. Our aim was to assess whether cervical cerclage in these patients was associated with prolonged pregnancy latency.

**Methods:**

This was an observational study of asymptomatic singleton pregnancies without a history of spontaneous preterm birth with an extremely short cervix (TVCL ≤ 10 mm) in the second trimester. Exposure and outcome data were extracted manually from medical charts by obstetric providers, with all outcomes occurring prior to study initiation. All patients were prescribed vaginal progesterone and those with a cervical dilation >1.5 cm were excluded. The primary outcome was time interval from diagnosis to delivery (or 37 weeks’ gestation, whichever occurred first). Secondary outcomes included gestational age at delivery, mode of delivery, and neonatal outcomes. We adjusted for parity and used log‐binomial regression to estimate the relative risk for categorical variables and linear regression to estimate mean differences for continuous variables.

**Results:**

There were 247 patients with TVCL ≤ 10 mm during anatomy scan. After exclusions, 87 remained, of which 55 (63.2%) received cervical cerclage. At the time of diagnosis, the mean cervical length was 5.8 mm (cerclage group) versus 7.4 mm (no cerclage group, difference: −1.6; 95% confidence interval [CI], −0.6 to −2.6) and the gestational age was 21.0 weeks (cerclage group) versus 22.1 weeks (no cerclage group, difference: −1.1; 95% CI, −1.8 to −0.3). Mean pregnancy latency was longer in the cerclage group compared to the no cerclage group (13.4 vs. 11.1 weeks, difference: 2.2; 95% CI, −0.3 to 4.9), though there was a high level of uncertainty in the estimate. Term delivery occurred 30% more often in the cerclage group compared to the no cerclage group (relative risk, 1.31; 95% CI, 0.96–1.79).

**Conclusion:**

In our cohort, patients with extremely short cervix who received a cerclage had longer pregnancy latency than those who did not receive cerclage and more patients achieved term gestation, suggesting a potential benefit of cerclage in this population. However, given the high level of uncertainty of our estimates, additional research is needed to investigate these findings.

## 1 INTRODUCTION

Placement of a cervical cerclage significantly decreases the risk of spontaneous preterm birth (PTB) in several clinical scenarios, such as in patients with a prior history of cervical insufficiency or painless cervical dilation in the second trimester [1]. While the benefit of cerclage in the setting of transvaginal cervical length (TVCL) ≤ 25 mm is well established for those with a prior history of spontaneous PTB, the effect in those without this history is less clear [2].

Vaginal progesterone has been the cornerstone for the prevention of PTB for asymptomatic patients in the mid‐trimester with short cervix, singleton pregnancy, and no prior spontaneous preterm birth [1, 3]. Recently, there has been interest in the potential benefit of cerclage in certain subgroups of this population at highest risk, particularly those with short cervix. One recent multicenter retrospective cohort study found that the risk of spontaneous PTB was lower at all gestational ages for patients without a history of spontaneous PTB who were treated with cerclage and vaginal progesterone compared with cerclage alone for cervical length (CL) <10 mm or cervical dilation [4]. A recent multicenter randomized controlled trial comparing cerclage in addition to vaginal progesterone vs. progesterone alone for the management of a short cervix (≤25 mm CL on transvaginal ultrasound) in singleton gestations before 24 weeks without a prior spontaneous PTB found increased pregnancy latency from randomization to delivery and increased gestational age at delivery [5].

In light of these recent findings, updated guidelines from American College of Obstetricians and Gynecologists and Society of Maternal‐Fetal Medicine now state that cerclage can be “considered” in patients with TVCL <10 mm and without a history of spontaneous PTB, based on shared decision‐making [1, 3]. To inform shared decision‐making, additional evidence is needed to further characterize the risks and benefits in this subpopulation. The aim of this study is to evaluate whether cerclage is associated with increased pregnancy latency in patients with an extremely short cervix (TVCL ≤ 10 mm) with no history of spontaneous PTB. We hypothesized that cerclage placement would be associated with an increase in latency and improved neonatal outcomes.

## 2 MATERIALS AND METHODS

We conducted an observational cohort study of singleton pregnancies with a TVCL ≤ 10 mm detected during universal TVCL screening on routine scan between 16‐ and 23‐weeks’ and 6 days’ gestation at three academic centers from January 2016 to June 2024. Exposure and outcome data were extracted manually from medical charts by an obstetric provider retrospectively, with all outcomes occurring prior to study initiation. Subjects were excluded if they had a history of spontaneous PTB between 16‐ and 34‐weeks’ gestation, cervical cerclage in prior pregnancy, major fetal structural anomalies, cervical surgeries (i.e., loop electrosurgical excision procedure and/or cold knife cone biopsy), suspected Mullerian anomalies, or history of gonorrhea and/or chlamydia in the pregnancy. We also excluded patients with symptoms associated with preterm labor, such as uterine contractions, abdominal or lower back pain, pelvic pressure, vaginal bleeding, or leakage of amniotic fluid or cervical dilation >1.5 cm as patients with greater cervical dilation are more similar to the population undergoing physical exam‐indicated cerclage for dilated cervix [5]. Patients without delivery data (whether due to pregnancy termination or delivery outside our health system) were also excluded. All patients included in this study were prescribed vaginal progesterone immediately after their diagnosis of an extremely short cervix, as reflected in their electronic medical records. Patients are counseled to continue taking vaginal progesterone until 36 weeks’ gestation, regardless of cerclage placement.

We compared time interval from initial diagnosis of extremely short cervix ≤10 mm to delivery or 37 weeks’ gestation, whichever occurred first, between those who received a cerclage and those who did not. Secondary outcomes include gestational age at delivery stratified by various preterm gestational age ranges (i.e., PTB <24 weeks, <28 weeks, <34 weeks, <37 weeks, and ≥37 weeks), mode of delivery, admission to the neonatal intensive care unit (NICU), birthweight, fetal demise, and neonatal demise within 30 days of birth. Other secondary outcomes included procedure‐related complications such as preterm pre‐labor rupture of membranes (PPROMs) at the time of placement or cervical bleeding with placement.

Data on maternal characteristics including age, body mass index (BMI; kg/m^2^), parity, chronic hypertension, pregestational diabetes, and history of cesarean section were also retrospectively collected via manual chart review performed by clinicians. Given previously reported disparities in the rates of PTB associated with race and ethnicity, self‐identified race and ethnicity were also collected. Gestational age, CL, and cervical dilation at the time of diagnosis of an extremely short cervix were also collected.

Within our health system, we perform universal CL screening using transvaginal ultrasound during second trimester fetal anatomical survey, which is typically performed at 18–22 weeks’ gestation. Our standardized protocol includes taking three measurements with an empty maternal bladder. Sonographers magnify the cervix to approximately two thirds of the screen; anatomic landmarks are identified including the internal and external ossa and endocervical canal, as well as the width of the anterior and posterior lips of the cervix. Trans‐fundal pressure is typically employed to identify dynamic changes, though CL measurements are not assessed concurrently. The functional or shortest CL is recorded. These measurements are performed by registered diagnostic medical sonographers and reviewed by Maternal‐Fetal Medicine specialists.

Patients diagnosed with an extremely short cervix ≤10 mm are counseled about the findings in the outpatient ultrasound unit. All patients included in this study were recommended vaginal progesterone. The decision to proceed with cerclage versus continue outpatient surveillance of the cervix was determined by the provider in conjunction with the patient. If cerclage was recommended, patients were referred to Labor and Delivery for placement within 24 h. All patients had cervical exam performed and documented prior to cerclage placement. Cerclage was not offered after 24 weeks’ and 0 days’ gestational age. Cervical cerclage surgical approach, suture type, and use of preoperative antibiotic or postoperative indomethacin treatment were determined by the managing provider. The cervical cerclages were performed by placing a transvaginal purse‐string suture at the level of the internal os at the cervicovaginal junction (McDonald technique) or after displacement of both bladder and rectum (Shirodkar technique) or at the cervicovaginal junction [6, 7].

Comparisons between groups were evaluated through the mean differences and 95% confidence intervals (CIs) for covariates or outcome variables and through the relative risk for categorical covariates and outcomes. The relative risk was estimated through modified Poisson regression models and outcomes were adjusted for parity [8]. We intentionally have avoided significance testing with *p* values due to concerns that have been raised by organizations such as the American Statistical Association [9] about dichotomizing research findings into “statistically significant” or “not statistically significant.” Instead, we emphasize effect sizes and CIs to shed light on the robustness of associations [4, 10]. The time to delivery was estimated as the time from ultrasound scan to delivery and Kaplan–Meier curves were used to compare median delivery times by cerclage group. Analysis of time to delivery examined how cerclage intervention impacted latency of pregnancy. In a prespecified subgroup analysis, we repeated these comparisons among patients with closed cervix with a more conservative cutoff focusing on patients with closed cervix on digital examination, excluding those patients with cervical dilation more than fingertip (> 0.5 cm). All statistical analyses were performed using SAS v9.4. This study was approved by the NYU Institutional Review Board (i24‐00434) under a waiver of informed consent.

## 3 RESULTS

**FIGURE 1:**
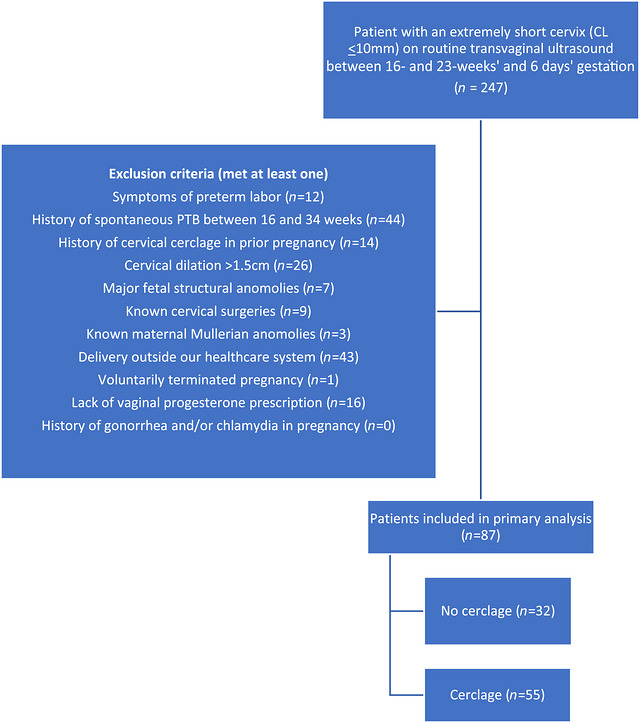
Study population. CL, cervical length; PTB, preterm birth.

From January 2016 to June 2024, a total of 247 patients were diagnosed with an extremely short cervix ≤10 mm, which we estimate represents approximately 1% of all patients screened for CL at the three hospital systems during the study period. After the exclusion criteria were applied, 87 patients were included in the study cohort (Figure 1). The most common reasons for exclusion were a history of prior spontaneous PTB (*n* = 44) and delivery outside our hospital system (*n* = 42).

Among the 87 eligible patients, 55 (63.2 %) underwent cervical cerclage and 32 (36.8%) did not undergo cerclage (Table 1). At the time of diagnosis, the gestational age was 21.0 (cerclage group) versus 22.1 weeks and mean CL was 5.8 (SD: 2.3) mm (cerclage group) versus 7.4 (SD: 2.5) mm (no cerclage group, mean difference in cervical length −1.6 [95% CI, −2.6 to −0.6], Table 1). The cerclage group had higher average BMI (30.9 [SD: 6.3] kg/m^2^ vs. 26.2 [SD: 4.8] kg/m^2^) and a higher proportion of patients who self‐identified as Black (47.3% vs. 18.8%) compared to the group that did not receive a cerclage (Table 1). Of the 55 patients who received cerclage 40 (73%) received antibiotics and 33 (60%) received tocolytics at the time of cerclage placement, administered at the treating team's discretion. There were no immediate complications of cerclage placement, including PPROM or cervical hemorrhage.

**TABLE 1:** Baseline characteristics of the study cohort.

Baseline characteristic	No cerclage (*n* = 32)	Cerclage (*n* = 55)	Mean difference or relative risk (95% CI)	Adjusted mean difference or relative risk (95% CI)
Maternal age (years), mean (SD)	32 (6.3)	33.1 (5.7)	1.2 (−1.5, 3.8)	0.7 (−2.0, 3.4)
BMI (kg/m^2^), mean (SD)	26.2 (4.8)	30.9 (6.3)	4.8 (2.2, 7.3)	4.7 (2.1, 7.4)
Race, *n*(%)				
Asian	4 (12.5%)	6 (10.9%)	0.94 (0.55, 1.61)	0.92 (0.55, 1.54)
Black/African‐American	6 (18.8%)	26 (47.3%)	1.54 (1.14, 2.08)	1.55 (1.15, 2.07)
White	11 (34.4%)	11 (20%)	0.74 (0.47, 1.16)	0.73 (0.47, 1.13)
Other/Unknown	11 (34.4%)	12 (21.8%)	0.78 (0.51, 1.19)	0.79 (0.50, 1.22)
Ethnicity, *n*(%)				
Hispanic	4 (12.5%)	8 (14.5%)	1.06 (0.69, 1.65)	1.11 (0.74, 1.66)
Non‐Hispanic	26 (81.3%)	44 (80%)	0.97 (0.65, 1.44)	0.93 (0.64, 1.34)
Unknown	2 (6.3%)	3 (5.5%)	NA	NA
Nulliparous, *n*(%)	20 (62.5%)	44 (81.5%)	1.51 (0.93, 2.46)	NA
Chronic hypertension, *n*(%)	0 (0%)	6 (11.1%)	NA	NA
Pregestational diabetes, *n*(%)	2 (6.3%)	1 (2%)	0.52 (0.10, 2.62)	0.48 (0.10, 2.37)
History of cesarean section, *n*(%)	3 (9.4%)	3 (5.5%)	0.78 (0.34, 1.76)	1.14 (0.43, 3.03)
Mean gestational age at time of diagnosis (weeks), mean (SD)	22.1 (1.3)	21.0 (1.8)	−1.1 (−1.8, −0.3)	−1.0 (−1.8, −0.3)
Mean cervical length (mm), mean (SD)	7.4 (2.5)	5.8 (2.3)	−1.6 (−2.6, −0.6)	−0.2 (−0.3, −0.1)
Mean cervical dilation prior to cerclage (cm), mean (SD)	–	0.39 (0.48)	NA	NA

Abbreviations: BMI, body mass index; CI, confidence interval; NA, not applicable; SD, standard deviation.

After a diagnosis of extremely short cervix, we observed that the cerclage group had longer mean pregnancy latency compared to those who did not receive cerclage (13.4 vs. 11.1 weeks). There was an adjusted mean difference (aMD) of 2.4 (95% CI, −0.3 to 5.0) more weeks of latency for patients who received cerclage, adjusted for parity (Table 2). The mean gestational age at delivery was 34.4 weeks in the cerclage group compared to 32.8 weeks in the no cerclage group (aMD, 1.8; 95% CI, −1.1 to 4.7) (Table 2). There was 30% increased risk of term birth compared to birth <37 weeks with cerclage (adjusted relative risk [aRR], 1.31; 95% CI, [0.96–1.79]). Of the no cerclage group, 78.1% had a vaginal delivery, whereas 50.9% of the cerclage group had a vaginal delivery (aRR, 0.71; 95% CI, 0.51–0.98) (Table 2). Median time to delivery for the cerclage group was 15.6 (95% CI, 12.7–16.7) weeks compared to 12.0 (95% CI, 8.7–15.3) weeks in the no cerclage group. No association was observed between receipt of cerclage and NICU admission, fetal demise, or neonatal demise. A subgroup analysis evaluated outcomes for patients with a short TVCL ≤ 10 mm and a closed cervix. The test for interaction between cerclage and the subgroup on latency was non‐significant (*p* value, 0.105; parameter estimate, −4.5; 95% CI, −9.8 to 0.9).

**TABLE 2:** Primary and secondary outcomes of the study cohort.

Outcome	No Cerclage (*n* = 32)	Cerclage (*n* = 55)	Mean difference or relative Risk (95% CI)	aMD or aRR^a^ (95% CI)
Time interval to delivery or 37 weeks, mean (SD)	11.1 (6.4)	13.4 (5.4)	2.3 (−0.3, 4.9)	2.4 (−0.3, 5.0)
Gestational age at delivery (weeks), mean (SD)	32.7 (7.1)	34.4 (6.0)	1.6 (1.2, 4.5)	1.8 (−1.1, 4.7)
PTB at <24 weeks, *n*(%)	5 (15.6%)	4 (7.3%)	0.68 (0.32, 1.44)	0.71 (0.34, 1.47)
PTB at <28 weeks, *n*(%)	9 (28.1%)	11 (20%)	0.84 (0.54, 1.29)	0.80 (0.53, 1.23)
PTB at <34 weeks, *n*(%)	15 (46.9%)	20 (36.4%)	0.85 (0.60, 1.20)	0.82 (0.59, 1.14)
PTB at <37 weeks, *n*(%)	20 (62.5%)	25 (45.5%)	0.78 (0.56, 1.08)	0.76 (0.56, 1.04)
Term (≥37 weeks), *n*(%)	12 (37.5%)	30 (54.6%)	1.39 (0.93, 1.78)	1.31 (0.96, 1.79)
Vaginal delivery, *n*(%)	25 (78.1%)	28 (50.9%)	0.77 (0.63, 0.94)	0.71 (0.51, 0.98)
Neonatal intensive care unit admission, *n*(%)	13 (40.6%)	22 (40%)	0.99 (0.71, 1.37)	0.97 (0.71, 1.33)
Birthweight (g), mean (SD)	2349 (1092.7)	2464.4 (1176.4)	115.4 (−415.7, 646.5)	194.2 (−356.1, 744.4)
Fetal demise, *n*(%)	3 (9.4%)	2 (3.6%)	0.62 (0.21, 1.83)	0.63 (0.22, 1.84)
Neonatal demise, *n*(%)	2 (6.3%)	2 (3.6%)	0.78 (0.29, 2.11)	0.84 (0.32, 2.19)

Abbreviations: aMD, adjusted mean difference; aRR, adjusted relative risk; CI, confidence; PTB, preterm birth; SD, standard deviation.

^a^ Adjusted for parity.

## 4 DISCUSSION

We observed longer pregnancy latency in patients who received a cervical cerclage compared to no cerclage in asymptomatic singleton pregnancies with an extremely short cervix. It is important to note, however, that while the point estimates of association between cerclage and latency or PTB may suggest benefit, the CIs are wide, meaning that the possibility of no effect or even harm cannot be dismissed. These data suggest the need for a randomized trial to determine whether cerclage placement is of benefit in this high‐risk population.

Our findings build on prior studies demonstrating a potential role for cerclage in asymptomatic patients with extremely short cervix and no prior spontaneous PTB. Prior literature has found that for patients with progressively shortening CL to <10 mm while on vaginal progesterone, cervical cerclage placement significantly decreases rates of PTB <37 weeks and confers a twofold pregnancy latency prolongation [10]. However, patients with a CL <10 mm on their initial transvaginal ultrasound or with cervical dilation were excluded from this study, limiting its generalizability. Our study identified a potential benefit of cerclage in patients with a CL ≤ 10 mm at any time between 16‐ and 23‐weeks’ gestation, which increases the generalizability [11]. A subgroup analysis within a meta‐analysis found that cerclage placement was associated with significantly lower rates of PTB <35 weeks with CL <10 mm [12]. However, the quality of evidence in this meta‐analysis was low given small sample sizes and few outcome events. Our study addresses these limitations by including data from 3 sites with similar practice patterns and a substantial sample size. Finally, while other studies demonstrated increased pregnancy latency with cerclage in an extremely short cervix (≤10 mm), they were limited by small sample size and lack of universal assessment of cervical dilation, which calls into question whether several of these patients also met criteria for an exam‐indicated cerclage [10, 11]. By including a strict criteria for cervical dilation, we ensured more homogeneity and included a population that could benefit from cerclage that is currently not receiving this intervention routinely.

In our study, our cerclage group was more likely to be nulliparous and self‐identifies as Black compared to those who did not undergo cerclage. This could be because these characteristics are associated with an increased probability of PTB, which may have influenced the provider's recommendation to pursue a more aggressive intervention [13, 14]. This may suggest the possibility of selection bias, whereby those with higher pretest probability of spontaneous PTB received cerclage compared to those who were at lower risk. However, this would imply that the reduced incidence of PTB in the cerclage group (enriched in high‐risk patients) is that much more compelling.

Patients who received a cervical cerclage were more likely to deliver by cesarean delivery in our primary analysis. Prior literature on the association between cerclage and mode of delivery is heterogenous, with some groups finding no difference on the rates of cesarean section with cerclage placement and other groups finding a higher risk of cesarean section among patients with cerclage compared to no cerclage [11, 12, 15]. Further research is needed to better characterize the impact of cervical cerclage on the mode of delivery.

Our study has several strengths, including strict exclusion criteria to specifically evaluate the role of cerclage in asymptomatic patients with extremely short cervix. We ensured all patients were prescribed vaginal progesterone, which is the current standard of care [1, 3]. We included data from three sites with similar practice patterns but varying patient populations, which increases generalizability. We opted for an intention to treat approach to our analysis where we did not differentiate between preterm deliveries secondary to spontaneous PTB and indicated preterm deliveries for other conditions, similar to prior studies on cerclage. Given exposure and outcome data were extracted retrospectively, we are unable to assess causality directly and there is the possibility of unmeasured confounders. Our subgroup analysis interaction test for patients with a closed cervix was non‐significant, demonstrating that cervical dilation did not alter the findings, although the study may be underpowered to show a difference if it were to exist. We did not collect data on the indication for cesarean section, which limits our ability to comment on the association between cerclage placement and mode of delivery. Additional limitations of our study include small sample size and possible selection bias introduced from various practice patterns by providers, but we hope our data will be valuable in supporting and informing a randomized control trial or additional observational studies to further investigate this important question [16].

## 5 CONCLUSION

Our findings suggest that cervical cerclage placement in addition to vaginal progesterone in singleton pregnancies with an extremely short cervix and no history of spontaneous PTB may be associated with a longer pregnancy latency compared to management with vaginal progesterone alone. These data underscore the need for a trial to better characterize the benefits of cerclage placement in this high‐risk population.

## CONFLICT OF INTEREST STATEMENT

The authors declare no conflicts of interest.
