# Correction to “Cerclage for singleton pregnancies with an extremely short cervix (≤10 mm) and no history of spontaneous preterm birth: A multisite observational study”

**DOI:** 10.1002/pmf2.70313

**Published:** 2026-05-15

Kansal, N., M., Lantigua‐Martinez, S., Friedman, S.,Khurana, C., Goldberger, E.M., Hade, J., Silverstein, et al. (2026), Cerclage for Singleton Pregnancies With an Extremely Short Cervix (≤10 mm) and no History of Spontaneous Preterm Birth: A Multisite Observational Study. *Pregnancy*, 2: e70262. https://doi.org/10.1002/pmf2.70262.

The Funding Information section of this paper erroneously stated that the authors received no specific funding for this work. It should instead have read, “This work was supported in part by the National Institutes of Health under award number K08HD111687‐02.”

We apologize for this omission.

